# Survival of patients treated with intra-aortic balloon counterpulsation at a tertiary care center in Pakistan – patient characteristics and predictors of in-hospital mortality

**DOI:** 10.1186/1471-2261-4-22

**Published:** 2004-12-01

**Authors:** Fahim H Jafary, Sohail A Khan, Haresh Kumar, Numaan F Malik, Khawar A Kazmi, Sajid Dhakam, Azam Shafquat, Aamir Hameed, Javed Tai, Najaf Nadeem

**Affiliations:** 1 Department of Medicine, Section of Cardiology, Aga Khan University Hospital, Karachi 74800, Pakistan; 2 Department of Cardiology, National Institute of Cardiovascular Disease, Karachi, Pakistan

## Abstract

**Background:**

Intra-aortic balloon counterpulsation (IABC) has an established role in the treatment of patients presenting with critical cardiac illnesses, including cardiogenic shock, refractory ischemia and for prophylaxis and treatment of complications of percutaneous coronary interventions (PCI). Patients requiring IABC represent a high-risk subset with an expected high mortality. There are virtually no data on usage patterns as well as outcomes of patients in the Indo-Pakistan subcontinent who require IABC. This is the first report on a sizeable experience with IABC from Pakistan.

**Methods:**

Hospital charts of 95 patients (mean age 58.8 (± 10.4) years; 78.9% male) undergoing IABC between 2000–2002 were reviewed. Logistic regression was used to determine univariate and multivariate predictors of in-hospital mortality.

**Results:**

The most frequent indications for IABC were cardiogenic shock (48.4%) and refractory ischemia (24.2%). Revascularization (surgical or PCI) was performed in 74 patients (77.9%). The overall in-hospital mortality rate was 34.7%. Univariate predictors of in-hospital mortality included (odds ratio [95% CI]) age (OR 1.06 [1.01–1.11] for every year increase in age); diabetes (OR 3.68 [1.51–8.92]) and cardiogenic shock at presentation (OR 4.85 [1.92–12.2]). Furthermore, prior CABG (OR 0.12 [0.04–0.34]), and in-hospital revascularization (OR 0.05 [0.01–0.189]) was protective against mortality. In the multivariate analysis, independent predictors of in-hospital mortality were age (OR 1.13 [1.05–1.22] for every year increase in age); diabetes (OR 6.35 [1.61–24.97]) and cardiogenic shock at presentation (OR 10.0 [2.33–42.95]). Again, revascularization during hospitalization (OR 0.02 [0.003–0.12]) conferred a protective effect. The overall complication rate was low (8.5%).

**Conclusions:**

Patients requiring IABC represent a high-risk group with substantial in-hospital mortality. Despite this high mortality, over two-thirds of patients do leave the hospital alive, suggesting that IABC is a feasible therapeutic device, even in a developing country.

## Background

Intra-aortic balloon counterpulsation (IABC) has an established role in the treatment of patients presenting with cardiogenic shock [1-3], refractory heart failure [4,5], ischemia [6] and arrhythmias [7] as well as for prophylaxis [8,9] and treatment of complications of percutaneous coronary intervention (PCI). Patients requiring IABC represent a high-risk subset with an expected high mortality [10]. In an international registry of over 16,000 cases selected from primarily developed nations [11], the overall adjusted in-hospital mortality was 21.2%. However, there were geographic differences with lower mortality rates in U.S. patients compared to their non-US counterparts (20.1% vs. 28.7%; p < 0.001) [12]. Major predictors of mortality in these patients include age, gender, and presentation with cardiogenic shock. There is paucity of data on the usage patterns as well as outcomes of patients undergoing IABC in the Indo-Pakistan region. This is partly due to the limited availability and capacity to implant the device as only a few centers in Pakistan have the required logistical as well as technical expertise. Our institution has previously reported on our initial experience of 15 patients undergoing IABC prior to coronary artery bypass graft (CABG) surgery [13]. We now report on an extended experience with intra-aortic balloon counterpulsation and describe the patterns of usage as well as the independent predictors of in-hospital mortality in patients undergoing IABC.

## Methods

### Patient population

We reviewed the charts of 95 patients undergoing IABC at the Aga Khan University Hospital (AKUH), Karachi, Pakistan between January 2000 and December 2002. Patients requiring IABC in the operating room immediately following CABG to assist weaning off cardiopulmonary bypass were excluded from this study. However, those patients who underwent IABP implantation prior to surgery were included. The AKUH is a tertiary care hospital located in the metropolitan city of Karachi that receives a mixture of affluent as well as low and middle income patients and serves the entire city as a referral center for patients requiring high-intensity tertiary care. Variables collected included age, gender, indication for IABC (shock or non-shock), history of diabetes, hypertension, smoking, prior PCI or CABG, left ventricular function, refractory ischemia and treatment (revascularization vs. no revascularization). Cardiogenic shock was defined as a systolic blood pressure (SBP) of < 90 mm Hg for at least 30 minutes (or requirement of inotropes to maintain a SBP > 90 mm Hg) associated with hypoperfusion (decreased urine output or cool extremities) and a heart rate of ≥ 60 beats per minute. Left ventricular (LV) ejection fraction (EF) was assessed by visual estimation. LV function was recorded as normal for an EF of ≥ 55%, mildly impaired for an EF 40–54%, moderately impaired for an EF 26–39% and severely impaired if the EF was ≤ 25%. Heart failure was diagnosed using clinical signs as defined by the Framingham criteria [14]. Refractory heart failure was defined as heart failure failing to respond to therapy including inotropic support. Refractory ischemia was defined as on-going ischemic chest pain and/or dynamic ECG changes (ST depression or ST elevation ≥ 1 mm in two or more contiguous leads) despite adequate medical therapy including antiplatelet drugs, beta-blockers and heparin. The outcome of interest was in-hospital mortality.

### Statistical methods

All variables were entered into Statistical Package for Social Sciences (SPSS) version 10. Means and standard deviations were calculated for continuous variables and frequencies for categorical variables. Variables were analyzed by simple logistic regression to calculate the unadjusted odds ratios for factors associated with in-hospital mortality. Those variables with a p value of ≤ 0.25 on univariate analysis were entered into the multivariable model and adjusted odds ratios for factors associated with in-hospital mortality were calculated. Finally, the model fit was assessed using the Hosmer-Lameshow test. A p value of < 0.05 was considered significant.

## Results

Table 1 summarizes the patient characteristics. The mean age of the study group was 58.8 (± 10.4) years. The majority of subjects were male (78.9%) and a high proportion had hypertension (55.8%), diabetes (43.2%), a smoking history (37.9%), previous PCI (30.5%) or CABG (48.4%). About half (48.4%) of the patients presented with cardiogenic shock and a similar number (52.6%) had moderate or severe depression of left ventricular function at presentation. All except two patients underwent coronary angiography and over two-thirds had three-vessel coronary artery disease. A revascularization procedure (either surgical or PCI) was performed in 74 patients (77.9%). In the remaining 21 patients, the main reasons for not performing revascularization were as follows: diffuse disease not amenable to PCI or CABG (5 patients), CABG felt to be too high-risk on account of comorbid conditions (6 patients), death in the catheterization laboratory prior to revascularization (6 patients), failed PCI (1 patient) and no need for revascularization (3 patients). The overall in-hospital mortality rate in this study group was 34.7% with six patients (6.3%) dying in the laboratory while the remaining 27 (28.4%) died during the hospital stay. Sixty-five patients (65.3%) left the hospital alive.

**Table 1:** Patient Characteristics.

**Characteristic**	N (%)*
Age (mean/SD)	58.8 (10.4)
Males	75 (78.9)
Female	20 (21.1)
Diabetes	41 (43.2)
Hypertension	53 (55.8)
Smoking	36 (37.9)
Previous PCI	29 (30.5)
Previous CABG	46 (48.4)
Coronary Anatomy	
Single vessel disease	6 (6.3)
2-vessel disease	15 (15.8)
3-vessel disease	72 (75.8)
LV function – normal or mildly impaired	45 (47.4)
LV function – moderate or severely impaired	50 (52.6)
Cardiogenic shock	46 (48.4)
Underwent revascularization	74 (77.9)
Percutaneous	26 (27.4)
Surgical	48 (50.5)

* mean/Standard Deviation for age; (%) for others

LV = left ventricular; PCI = percutaneous coronary intervention; CABG = coronary artery bypass grafting

Table 2 shows the indications for the implantation of an intra-aortic balloon pump (IABP). Almost half were inserted for cardiogenic shock. In the univariate analysis (Table 3), variables associated with in-hospital mortality included (odds ratio [95% CI]) age (OR 1.06 [1.01–1.11] for every year increase in age; diabetes (OR 3.68 [1.51–8.92]) and cardiogenic shock at presentation (OR 4.85 [1.92–12.2]); left ventricular dysfunction, hypertension and 3-vessel (versus no 3-vessel) coronary artery disease were not significantly associated with in-hospital mortality in these patients. A significant protective effect of a prior history of CABG surgery (OR 0.12 [0.04–0.34]) and in-hospital revascularization, either surgical or percutaneous, (OR 0.05 [0.01–0.189]) was noted in this study.

**Table 2:** Indications for Intra-aortic balloon counterpulsation

**Indication**	**N (%)**
Cardiogenic shock	22 (23.2)
Cardiogenic shock with mechanical complication	24 (25.3)
Left Main disease, no chest pain	9 (9.5)
Left Main disease, chest pain in laboratory	6 (6.3)
Refractory heart failure	8 (8.4)
Refractory Ischemia	23 (24.2)
Complication during PCI	2 (2.1)

PCI = percutaneous coronary intervention (includes abrupt closure, severe "no-reflow")

**Table 3:** Univariate Predictors of In-Hospital Mortality

	**Survived (%) (n = 62)**	**Died (%) (n = 33)**	**Unadjusted OR (95% CI)**	**p value**
Age (SD)	56.9 (10.1)	62.5 (10.3)	1.06 (1.01–1.11) *	0.016
Male Gender	51 (82.3)	24 (72.7)	0.58 (0.21–1.57)	0.281
Diabetes	20 (32.3)	21 (63.6)	3.68 (1.51–8.92)	0.004
Hypertension	32 (51.6)	21 (63.6)	1.64 (0.69–3.93)	0.263
Smoking	25 (40.3)	11 (33.3)	1.35 (0.56–3.27)	0.504
Previous PCI	20 (32.3)	9 (27.3)	0.79 (0.31–2.0)	0.616
Previous CABG	40 (64.5)	6 (18.2)	0.12 (0.04–0.34)	<0.001
Cardiogenic Shock	22 (35.5)	24 (72.7)	4.85 (1.92–12.2)	0.001
3-vessel disease**	44 (72.1)	28 (87.5)	2.70 (0.83–8.89)	0.101
LV dysfunction ***	30 (48.4)	20 (60.6)	1.64 (0.70–3.87)	0.258
Revascularized	58 (95.1)	16 (48.5)	0.05 (0.01–0.19)	< 0.001

SD = standard deviation. PCI = percutaneous coronary intervention. CABG = coronary artery bypass graft. LV = left ventricular

* for every 1 year increase in age

** vs. no 3-vessel disease

*** moderate/severely impaired LV function vs. normal/mildly impaired

In the multivariate analysis (Table 4), the significant independent predictors of in-hospital mortality were age (OR 1.13 [1.05–1.22] for every year increase in age); diabetes (OR 6.35 [1.61–24.97]) and cardiogenic shock at presentation (OR 10.0 [2.33–42.95]). Revascularization during hospitalization remained a significant protective factor against mortality (OR 0.02 [0.003–0.12]) The Hosmer-Lemeshow test indicated a good fit for the model (χ^2 ^6.09; p = 0.637). In the adjusted analysis, a prior history of CABG did not remain a significant predictor of survival primarily because forty-five out of 46 patients underwent revascularization.

**Table 4:** Multivariate Predictors of In-hospital Mortality*

	**Survived (%) (n = 62)**	**Died (%) (n = 33)**	**Adjusted OR (95% CI)**	**p value**
Age (SD)	56.9 (10.1)	62.5 (10.3)	1.13 (1.05–1.22) *	0.001
Diabetes	20 (32.3)	21 (63.6)	6.35 (1.61–24.97)	0.008
Cardiogenic Shock	22 (35.5)	24 (72.7)	10.0 (2.33–42.95)	0.002
Revascularized	58 (95.1)	16 (48.5)	0.02 (0.003–0.12)	< 0.001

* adjusted for gender, previous CABG, hypertension and LV dysfunction (none/mild vs. moderate/severe). Hosmer-Lemeshow χ^2 ^6.09; p = 0.637

When age as a risk factor was further analyzed by plotting an ROC curve, an age cut-off of 66.5 years had a high specificity for the outcome of in-hospital mortality (specificity 83.9%; area under ROC-curve 0.66; p = 0.01). Thus older patients requiring IABC suffer worse outcomes than younger subjects.

The overall complication rate related to the device implantation was low. Eight patients (8.5%) developed limb ischemia necessitating removal of the IABP; however, only one of these eight required surgery. There were no significant bleeding complications although one patient developed a hematoma following removal of the device; however this patient did not require blood transfusion or surgical repair of the arteriotomy site.

## Discussion

Patients requiring IABC are at high risk for death on account of their critical underlying conditions. Despite this, several data have suggested that IABC can improve morbidity and mortality in specific subsets of patients including those presenting with cardiogenic shock. The use of IABC in developing countries is limited on account of lack of equipment as well as skilled personnel who can insert and manage the device. Ours is the first report on a sizeable experience with IABC from the Indo-Pakistan subcontinent. Our experience is similar to that of other centers in the West. We report a high in-hospital mortality rate in patients undergoing IABC (almost 35%). However, given that nearly half of the subjects had cardiogenic shock at presentation, this mortality rate is reasonably acceptable. Advanced age (over 66.5 years), diabetes and cardiogenic shock at presentation were strong independent predictors of in-hospital mortality, while revascularization (either surgical or PCI) was associated with high odds of survival. The latter finding is consistent with recently reported data from the IABP Benchmark Registry [15]. Of particular interest is the finding that patients with a prior history of CABG were more likely to survive, a finding driven by the fact that the majority underwent revascularization. This suggests that repeat revascularization of patients with a prior history of bypass surgery (a clearly high-risk subset) is not only feasible but also effective in a developing country setting. Our complication rates were acceptably low, supporting the feasibility of using IABC in our setting.

Several limitations of this study should be acknowledged. First, the sample size is fairly small and this is reflected in the relatively wide confidence intervals for the odds ratios. Due to a small sample size, it is difficult to make a comparison of correlates of mortality between subgroups, for example those presenting with cardiogenic shock versus those who did not and those undergoing surgical versus percutaneous revascularization. However, as expected, patients presenting with shock had a significantly higher mortality (72.7% vs. 27.3%; p = 0.001). Second, the patient group selected may not be representative of other centers in Pakistan given that our institution is a unique tertiary care hospital in the country. Third, our cohort did not contain patients undergoing prophylactic IABC prior to high-risk PCI for indications other than cardiogenic shock. This probably represents practice patterns at our institution whereby, largely due to cost constrains, very high-risk patients (for example those with multivessel disease and/or severe impairment of LV function) are preferentially send for surgery. The cumulative cost of IABC with multivessel stenting far exceeds that of a bypass operation. Only two patients required emergent IABC during PCI in the study period. This may reflect a selection of lower risk patients for PCI at our institution. Fourth, while the survival rate following IABC is nearly 65%, no analysis has been made of the cost effectiveness of this therapy.

## Conclusions

In conclusion, cardiogenic shock and refractory ischemia are common indications for IABC in a Pakistani setting. Patients requiring an IABP represent a high-risk group with substantial in-hospital mortality. This is consistent with the nature of the presenting illnesses in these patients and is similar to western data. Despite this high mortality, over two-thirds of patients do leave the hospital alive, suggesting that IABC is a feasible therapeutic device, even in a developing country. Age (particularly over 66.5 years), diabetes and cardiogenic shock at presentation are significant predictors of mortality in this group of patients. Revascularization is a significant predictor of survival and complication rates are acceptably low. Larger studies are needed to evaluate which subsets of patients benefit the most from this device and further cost effectiveness analyses are warranted.

## Competing interests

The author(s) declare that they have no competing interests.

## Authors' contributions

FHJ, SAK conceptualized this study and participated in the study design. FHJ performed the statistical analysis. HK, NFM collected the data. KAK, SD, AS were involved in manuscript review. AH, JT and NN participated in manuscript drafting and review. All authors have read and approved the final manuscript.

## Pre-publication history

The pre-publication history for this paper can be accessed here:



## References

[B1] Holmes DRJ (2003). Cardiogenic shock: a lethal complication of acute myocardial infarction. Rev Cardiovasc Med.

[B2] Bregman D, Casarella WJ (1980). Percutaneous intraaortic balloon pumping: initial clinical experience. Ann Thorac Surg.

[B3] Sanborn TA, Sleeper LA, Bates ER, Jacobs AK, Boland J, French JK, Dens J, Dzavik V, Palmeri ST, Webb JG, Goldberger M, Hochman JS (2000). Impact of thrombolysis, intra-aortic balloon pump counterpulsation, and their combination in cardiogenic shock complicating acute myocardial infarction: a report from the SHOCK Trial Registry. SHould we emergently revascularize Occluded Coronaries for cardiogenic shocK?. J Am Coll Cardiol.

[B4] Kuchar DL, Campbell TJ, O'Rourke MF (1987). Long-term survival after counterpulsation for medically refractory heart failure complicating myocardial infarction and cardiac surgery. Eur Heart J.

[B5] Kopman EA, Ramirez-Inawat RC (1980). Intra-aortic balloon counterpulsation for right heart failure. Anesth Analg.

[B6] Amsterdam EA, Awan NA, Lee G, Low R, Joye JA, Foerster J, Rendig S, Mason DT (1981). Intra-aortic balloon counterpulsation: rationale, application and results. Cardiovasc Clin.

[B7] Cowell RP, Paul VE, Ilsley CD (1993). The use of intra-aortic balloon counterpulsation in malignant ventricular arrhythmias. Int J Cardiol.

[B8] Anwar A, Mooney MR, Stertzer SH, Mooney JF, Shaw RE, Madison JD, VanTassel RA, Murphy MC, Myler RK (1990). Intra-aortic balloon counterpulsation support for elective coronary angioplasty in the setting of poor left ventricular function: a two center experience. J Invasive Cardiol.

[B9] Aguirre FV, Kern MJ, Bach R, Donohue T, Caracciolo E, Flynn MS, Wolford T (1994). Intraaortic balloon pump support during high-risk coronary angioplasty. Cardiology.

[B10] Stone GW, Ohman EM, Miller MF, Joseph DL, Christenson JT, Cohen M, Urban PM, Reddy RC, Freedman RJ, Staman KL, Ferguson JJ (2003). Contemporary utilization and outcomes of intra-aortic balloon counterpulsation in acute myocardial infarction: the benchmark registry. J Am Coll Cardiol.

[B11] Ferguson JJ, Cohen M, Freedman RJJ, Stone GW, Miller MF, Joseph DL, Ohman EM (2001). The current practice of intra-aortic balloon counterpulsation: results from the Benchmark Registry. J Am Coll Cardiol.

[B12] Cohen M, Urban P, Christenson JT, Joseph DL, Freedman RJJ, Miller MF, Ohman EM, Reddy RC, Stone GW, Ferguson JJ (2003). Intra-aortic balloon counterpulsation in US and non-US centres: results of the Benchmark Registry. Eur Heart J.

[B13] Jafarey AM, Amanullah M, Khan SA, Hasan SB (2000). The use of intra aortic baloon pump in patients undergoing coronary artery bypass grafting at the Aga Khan University Hospital, Karachi. J Pak Med Assoc.

[B14] McKee PA, Castelli WP, McNamara PM, Kannel WB (1971). The natural history of congestive heart failure: the Framingham study. N Engl J Med.

[B15] Urban PM, Freedman RJ, Ohman EM, Stone GW, Christenson JT, Cohen M, Miller MF, Joseph DL, Bynum DZ, Ferguson JJ (2004). In-hospital mortality associated with the use of intra-aortic balloon counterpulsation. Am J Cardiol.

